# Patterns of creative technique use in child and youth psychotherapy in Germany: an exploratory mixed-methods survey including identity-informed practice

**DOI:** 10.3389/frhs.2026.1824536

**Published:** 2026-07-07

**Authors:** Susanne Birnkammer, Briana Applewhite, Yuna Altmann, Ania Zubala, Morten L. Kringelbach, Claudia Calvano

**Affiliations:** 1 Clinical Child and Youth Psychology and Psychotherapy, Freie Universität Berlin, Berlin, Germany; 2 German Center for Mental Health (DZPG), Partner Site Berlin-Potsdam, Germany; 3 Department of Psychiatry, University of Oxford, Oxford, United Kingdom; 4 Centre for Eudaimonia and Human Flourishing, Oxford, United Kingdom; 5 Centre for Clinical Brain Sciences, College of Medicine and Veterinary Medicine, University of Edinburgh, Edinburgh, United Kingdom; 6 Aarhus University, Aarhus, Denmark

**Keywords:** art-based techniques, child and adolescent psychotherapy, racism-informed care, therapeutic alliance, transdiagnostic

## Abstract

**Background:**

Creative and arts-based techniques are increasingly integrated into child and adolescent psychotherapy, yet little is known about how they are applied in routine practice and whether their use differs when working with racially minoritized youth. This study examined the prevalence, clinical functions, and contextual positioning of art-based techniques among therapists in Germany, with a focus on identity-informed applications in work with clients of Color and clients from Black ethnic groups.

**Methods:**

This study reports data from the German arm of the CREATE project, an online cross-sectional mixed-methods research initiative conducted in Germany and the United Kingdom. Therapists working with children and adolescents (*N* = 98; Mean age = 36.60, SD = 9.84) completed quantitative items on use, goals, and perceived preparedness across client contexts (general vs. PoC vs. Black), and provided open-ended responses on strengths, challenges, and barriers. Quantitative analyses used descriptive statistics and non-parametric within-therapist comparisons; qualitative data were analyzed via framework analysis.

**Results:**

Overall, 62.2% of therapists reported using art-based techniques (51.0% exclusively; 11.2% combined with music-based methods). Use was most frequent in individual settings and was primarily described as transdiagnostic, targeting emotional access, engagement, nonverbal communication, and experiential processing; diagnosis-specific mentions were less common (most often anxiety, ADHD, depression, and trauma-related symptoms). Therapists with experience working with BIPoC clients and with non-binary clients were more likely to use art-based techniques. Within-therapist comparisons showed significantly lower perceived preparedness in PoC contexts, alongside reduced endorsement of art-based techniques for relationship-building and for supporting nonverbal communication of difficult-to-express experiences. Qualitative findings highlighted benefits for communication, emotional expression, access to avoided topics, and self-efficacy, while barriers centered on limited training, institutional constraints, and uncertainty about structuring and integrating techniques. Only 17.3% reported racism-informed training and 4.1% training on racial trauma.

**Conclusions:**

Art-based techniques are widely valued and used flexibly in German routine child and adolescent psychotherapy, predominantly as transdiagnostic, process-oriented tools. However, their positioning in minoritized contexts appears shaped by lower therapist preparedness and limited racism-informed training. Integrating structured training in creative techniques alongside culturally responsive and racism-informed psychotherapy may support more confident, relational, and identity-informed application in diverse youth populations.

## Introduction

In recent years, creative and arts-based approaches have gained increasing attention (1). Techniques such as art therapy, music therapy, dance therapy, and other expressive modalities are increasingly explored not only as supplements to traditional talking therapies, but also as distinct, multimodal interventions that support emotional regulation, therapeutic engagement, and identity development (2–4). Broadly defined, creative therapies encompass approaches that engage the imagination, body, and senses, often involving visual arts, music, movement, drama, or storytelling and providing symbolic, embodied, and nonverbal forms of expression (5, 6). These methods are particularly relevant for young clients who may struggle with verbal communication (2, 7) or who have experienced trauma (8), marginalization, or systemic discrimination (3). It is important to note that the present paper uses the term “art-based techniques” as a broad, inclusive umbrella to encompass three related but distinct levels of practice. Formal creative arts therapies (CATs) — such as art therapy or music therapy — refer to structured, professionally trained modalities delivered by credentialed specialists, in which the creative process itself is the primary therapeutic vehicle (1, 5). Psychotherapy-integrated creative techniques refer to the use of visual, musical, or expressive activities within a broader psychotherapeutic framework, where creative elements supplement or enhance an existing therapeutic approach such as CBT or psychodynamic therapy (2). Expressive interventions, finally, refer to more informal or spontaneous uses of creative expression — such as drawing, storytelling, or role play — that may be introduced opportunistically within sessions to facilitate communication or engagement, without a structured therapeutic rationale. While these distinctions are clinically meaningful, they are not always clearly demarcated in routine practice, and the present study deliberately captures this full spectrum in order to reflect how creative methods are actually used across diverse child and youth psychotherapy settings in Germany.

One population for whom creative techniques may offer particular therapeutic value are racially minoritized children and youth (9, 10). BIPoC (Black, Indigenous, and People of Color) youth are at elevated risk for developing mental health difficulties such as anxiety, depression, and posttraumatic symptoms (11, 12). This heightened vulnerability seems to be associated with the consequences of ongoing systemic inequalities such as structural racism and experiences of discrimination and exclusion (13–15). Despite the urgent need for mental health support among BIPoC youth, systemic barriers continue to restrict their access to adequate treatment (16). When BIPoC clients do access care, they often encounter models that are not culturally responsive, reflect Eurocentric norms (17, 18), fail to resonate with their lived experiences and cultural realities (45), or, in some cases, may even be retraumatizing (19).

BIPoC clients often report lower levels of trust, cultural attunement, and therapeutic alliance in conventional psychotherapy settings, due to experiences of misattunement, racial stereotyping, and a lack of representation or cultural understanding (20, 21). Clients from racially minoritized backgrounds often report dissatisfaction with conventional psychotherapy when their therapists lack cultural attunement, minimize experiences of discrimination, or fail to address the dynamics of power and identity in the therapeutic relationship (22). Underlying this are broader systemic issues — including what DiAngelo (23) refers to as “White discomfort”, which can inhibit therapists from meaningfully engaging with race and identity in therapy. White discomfort not only limits open dialogue about race but can also reinforce clients' feelings of invisibility or invalidation within the therapeutic space (24). Creative methods may help bridge this gap by offering therapists alternative entry points into such topics, and by enabling clients to engage with complex identity-related themes in a less confrontational, more exploratory way (25). A recent meta-analysis found that creative arts based methods are effective methods for reducing trauma symptoms in Global Majority and non-Western populations, namely young people in West-Africa and Syrian and Palestinian refugees, and highlighted the culturally resonant functioning of creative arts in these populations (26). These findings are promising; however, the existing evidence base remains methodologically heterogeneous, with most studies reporting positive but inconclusive outcomes and calling for more rigorous research designs (27, 28). Furthermore, the majority of studies have been conducted in specific or crisis-affected contexts — such as with refugee youth or children with PTSD (26–28) — and evidence from routine psychotherapy contexts in European countries such as Germany remains limited. As such, while creative therapies may provide a more flexible, identity-affirming, and culturally attuned framework for addressing the needs of BIPoC youth, little is known about how these approaches are actually implemented in routine practice with racially minoritized youth in the German context.

Importantly, the use of creative methods in child and youth psychotherapy is not rare; many therapists already integrate elements of drawing, music, or symbolic play into their work to enhance engagement, or therapeutic alliance (2). However, despite their widespread use, little is known about how creative techniques are actually applied in day-to-day psychotherapeutic practice, particularly across diverse clinical settings and with marginalized populations. Existing research has largely focused on the efficacy of formalized creative arts therapies or the development of conceptual frameworks (1, 2), leaving a gap in understanding how such methods are applied in everyday therapy sessions. There is also a lack of knowledge about how therapist-level factors, such as training background, therapeutic perspectives, racial identity, or professional experience influence the use of creative modalities in child and youth psychotherapy (2).

The present study builds on three interconnected premises: (1) racially minoritized youth face heightened mental health burden and encounter specific barriers in conventional psychotherapy; (2) therapist-level factors, including training gaps and White discomfort, further limit the responsiveness of existing approaches; and (3) creative and art-based techniques may offer a more flexible, nonverbal, and culturally attuned alternative that addresses these gaps (see Supplementary Figure S1).

The CREATE project (Cross-national Research on Exploring Applications of Creative Therapeutic Experiences) examines how children and youth psychotherapists, including art and music therapists, in Germany and the UK use creative techniques in their work. This paper reports findings from the German sample, focusing on general usage patterns and identity-informed applications in work with racially minoritized youth. By surfacing practitioner perspectives, the study contributes to a more grounded understanding of how creative approaches are used in routine child and youth mental health care in Germany.

## Methods

### Study design

This study reports findings from the German arm of the CREATE project (Cross-national Research on Exploring Applications of Creative Therapeutic Experiences), an ongoing mixed-methods research initiative conducted in Germany and the United Kingdom. An online cross-sectional mixed-methods survey exploring therapists' use of creative techniques in child and youth psychotherapy was conducted in Germany between April and August 2025. The study combines quantitative descriptive analysis with qualitative content analysis of open-ended responses. Data were collected via an online questionnaire hosted on unipark. Therapists were invited to participate through professional mailing lists, flyer, psychotherapy training institution networks, social media, and snowball sampling.

### Participants

A total of *N* = 98 therapists (Mean age: 36.60 (*SD* = 9.84) participated in the German sample. The inclusion criteria were: (a) current or past work with children and/or adolescents in a therapeutic setting, and (b) training or practice in psychotherapy, art therapy, or music therapy. Importantly, therapists were eligible regardless of whether they used creative techniques in their work. The sample included licensed adult psychotherapists (*n* = 10, 11.2%) and in training (*n* = 8, 9.0%), licensed child and youth psychological psychotherapists (*n* = 28, 28.6%), and in training (*n* = 54, 60.7%), art therapists (*n* = 13, 13.3%), and other non-specified mental health professionals (*n* = 3, 3.4%). Only one participant reported practicing in the UK, with the remaining participants practicing in Germany. On average, participants had 4.9 years of professional experience. See Table 1 for the demographics of the participants.

**Table 1:** Demographic characteristics of participants (*N* = 98).

Category	Subcategory	*n* (%)
Population Density	Rural area	4 (4.1%)
	Small town	13 (13.3%)
	Medium-sized city	17 (17.3%)
	Large city	42 (42.9%)
	Metropolitan area	22 (22.4%)
Years of Experience	0 years	43 (44.8%)
	1–3 years	23 (24.0%)
	4–9 years	13 (13.5%)
	10 + years	17 (17.7%)
Annual Household Income	< €20,000	19 (19.4%)
	€20,001–40,000	32 (32.7%)
	€40,001–60,000	19 (19.4%)
	€60,001–80,000	14 (14.3%)
	€80,001–100,000	7 (7.1%)
	> €100,001	7 (7.1%)
Racial/Ethnic Identity	White	80 (81.6%)
	More than one racial/ethnic group	3 (3.1%)
	Person of Color	3 (3.1%)
	Prefer not to answer	3 (3.1%)
	Self-identified (open responses)*	9 (9.2%)
Ethnic/Racial Minority	Yes	12 (12.2%)
Gender Identity	Cis-female	82 (83.7%)
	Cis-male	13 (13.3%)
	Non-binary	1 (1.0%)
	Questioning	1 (1.0%)
	Inter	1 (1.0%)
Therapeutic Orientation	Cognitive Behavioral Therapy (CBT)	74 (83.1%)
	Systemic	8 (8.2%)
	Psychodynamic	11 (11.2%)
	Psychoanalysis	1 (1.1%)
	Humanistic	1 (1.1%)
	Eclectic-integrative	2 (2.2%)

Self-identified responses under “Racial/Ethnic Identity” included: “German, raised in a multicultural family,” “A woman who was born and is rooted in Germany,” “European,” “Light-skinned,” “I do not differentiate between groups. I reflect on this…,” “Southern European,” “Turkish,” and “White, but with a multicultural background through marriage (Southern European).” These entries were grouped under “Self-identified” to reflect the complexity and fluidity of racial/ethnic identity beyond predefined categories. Multiple responses were permitted for therapeutic orientation; therefore, percentages may exceed 100%. Valid responses for this item: *n* = 89.

### Study material

The questionnaire was developed collaboratively by the interdisciplinary research team and informed by prior literature on creative arts therapies (1, 2, 5), culturally responsive care (20, 24, 29) and psychotherapy practice with children and adolescents (2, 8). As no existing measure adequately captured the combined focus on creative technique use, identity-informed practice, and work with racialized client groups, most items were specifically developed for this exploratory study. To support content validity, survey items were iteratively discussed and reviewed within the research team for conceptual relevance, clarity, comprehensibility, and alignment with the study aims. The development process incorporated both clinical and research perspectives, including expertise in art therapy, music therapy, child and adolescent psychotherapy, and racism-informed practice. One item assessing therapist self-reflection on race and identity in therapy was adapted from Kaurin et al. (30).

The survey comprised four main sections: (1) demographics and professional context (e.g., therapeutic orientation, training status), (2) frequency and type of creative methods used (e.g., art-based, music-based), (3) experiences and adaptations in working with BIPoC clients, and (4) reflective questions on the perceived benefits and barriers related to creative techniques.

The survey included Likert-scale questions, multiple choice items, and free-text responses and took approximately 10–15 min to complete. An English version of the full codebook is available upon request. As the questionnaire was newly developed for this study, no formal psychometric validation or pilot testing within the target population was conducted prior to data collection. The instrument should therefore be considered exploratory, and future research is needed to establish its psychometric properties. Preliminary evidence of measurement quality is nonetheless reported in Supplementary Table S2. Internal consistency estimates for multi-item checklist scales ranged from *α* = .490 to *α* = .628 across client contexts, which is considered acceptable for heterogeneous exploratory checklists (31). For single-item measures, cross-context convergent validity coefficients were strong (rs = .670 to.902, all *p* < .001), supporting the consistency of these measures across general, PoC, and Black client contexts. Table 2 shows an overview about the covered topics and examples for the items.

**Table 2:** Overview of the questionnaire.

Section	Topics Covered	Example Items
1. Demographics & Professional Background	Age, gender identity, racial/ethnic identity, therapeutic orientation, training/licensure status, years of experience, income, urban/rural context, and country of practice	“What is your primary therapeutic approach?”,“How many years of experience do you have in therapeutic work with children/youth?”
2. Use of Creative Techniques *(separately for art- and music-based methods)*	Type and frequency of method use, target age groups, treatment goals, and perceived effectiveness	“Which of the following techniques have you used in your work with children and adolescents?”,“What goals do you typically address with these techniques?”
3. Work with Racialized Clients *(for art/music separately)*	Experience working with BIPoC clients, adaptations made to creative methods, confidence in culturally responsive care, and received training on racism-informed or anti-racist practice	“Have you worked with BIPoC children and adolescents in therapy?”,“Have you received training on racism-informed or racial trauma care?”
4. Specific Focus on Black Clients *(for art/music separately)*	Perceived fit of creative methods for Black clients, confidence levels, and challenges encountered	“How well do you think creative techniques fit the needs of Black clients?”,“How confident do you feel in adapting your approach for Black clients?”
5. Barriers, Institutional Support & Self-Reflection*(for art/music separately and for 3 client groups: all/general clients, PoC clients, Black clients)*	Barriers to creative method use, Supervision/training access, Final self-reflection on therapist identity and positioning	“What barriers have you encountered when using creative methods with PoC clients?”“How do you reflect on your own identity and social position in therapeutic work?”

All content related to art-based and music-based methods was presented separately, conditional on participants’ prior experience with each modality. Most items were developed by the research team. The final self-reflection item was adapted from Kaurin et al. (30). The first and second authors, respectively trained in art therapy and music therapy, contributed their disciplinary perspectives and co-led the survey design.

### Terminology and ethnic categorization

In the German survey, therapists were asked about their experience working with racially minoritized clients using the umbrella terms “BIPoC” (Black, Indigenous, and People of Color) and “Black ethnic groups.” These terms reflect terminology commonly used in contemporary German academic and professional discourse to describe racialized experiences. The categorization was intentionally broad in order to capture therapists' perceived work with minoritized youth across diverse backgrounds. Three participants self-identified as having a mixed ethnic background. Given the exploratory focus of the present study, a more detailed ethnic breakdown was not assessed.

### Data analysis

Quantitative data were analyzed using descriptive statistics in SPSS (version 29.0) (32); and R (version 4.5.2) (44). Frequencies and percentages were calculated for categorical variables, and means and standard deviations for continuous variables where appropriate.

To examine associations between therapist characteristics and the general use of art-based techniques (yes/no), bivariate analyses were conducted using chi-square tests for categorical predictors and Mann–Whitney *U*-tests for ordinal variables.

To investigate whether therapists evaluated or applied art-based techniques differently depending on client context, within-therapist paired comparisons were performed. As the rating variables were ordinal and not normally distributed, non-parametric tests were used. Wilcoxon signed-rank tests compared therapists' ratings (e.g., perceived preparedness, effectiveness, confidence, and engagement) between general and PoC client contexts. Changes in the motive and therapeutic aim of art-based activities (yes/no) across contexts were examined using McNemar tests. In an exploratory analysis, differences across three client contexts (general, PoC, and Black clients) were assessed using Friedman tests for related samples. Due to smaller subsample sizes, these analyses were interpreted cautiously and considered exploratory. Given the exploratory nature of this study and the number of bivariate tests conducted (*k* = 9), *p*-values were corrected for multiple comparisons using the Benjamini-Hochberg false discovery rate (FDR) procedure (33). All findings are interpreted as hypothesis-generating.

Qualitative responses to open-ended questions were analyzed using qualitative framework analysis (34). An initial coding framework was developed based on familiarization with the data and the study's research questions and was iteratively refined during analysis. Two independent raters (S.B. & Y.A.) then applied the framework to all responses. Because multiple codes could be assigned to a single response, inter-rater reliability was assessed using Cohen's kappa calculated across binary code assignments (code present vs. absent per segment). Across all coding sections, inter-rater agreement indicated moderate agreement between raters (*κ* = 0.537). Agreement levels varied across individual codes, ranging from fair to almost perfect agreement, with lower coefficients primarily observed for infrequently assigned or conceptually overlapping codes. Detailed inter-rater reliability coefficients for all coding sections and individual codes are provided in the Supplementary Materials. Following independent coding, discrepancies were discussed and resolved through consensus meetings, resulting in a fully agreed final coding dataset. Initial disagreements mainly concerned distinctions between structural barriers, therapist confidence, and therapeutic orientation.

Counts (n) are reported only for subgroup-specific mentions (e.g., PoC and Black clients) to illustrate response distribution. Percentages were not calculated because open-ended responses varied in length and number of coded statements per participant.

### Reflexivity and researcher positionality

The research team comprised members with diverse professional backgrounds, including training in art therapy, child and adolescent psychotherapy, and racism-informed practice. The two primary coders (S.B. and Y.A.) brought complementary perspectives to the analytic process: S.B. is a trained art therapist with clinical experience in child and youth psychotherapy, which supported a nuanced reading of practice-related accounts, while Y.A., though embedded in the same research unit (Clinical Child and Youth Psychology and Psychotherapy), approached the data without a specialized background in creative arts therapies, providing a more distanced and independent perspective. Both coders engaged in ongoing reflection on how their respective training and prior assumptions might shape interpretation, and discrepancies were discussed and resolved through consensus meetings. Furthermore, we acknowledge that our positionality as researchers embedded in a predominantly White, German academic context may have influenced how themes were conceptualized and prioritized, and we have aimed to remain reflexive about these limitations throughout the analytic process.

### Ethical considerations

The study was approved by the Ethics Committee of the Department of Education and Psychology at Freie Universität Berlin (Approval No.: r1 005/2025). as well as by the Ethics Committee at Oxford University (MS IDREC 1266701). All participants provided informed consent in accordance with the Declaration of Helsinki. While the survey was conducted anonymously, participants were asked to share sensitive demographic information, such as their racial, ethnic and gender-identity, age, and professional background. These data were collected in a non-identifiable format and stored securely in compliance with the European Union's General Data Protection Regulation (GDPR). Due to the potentially sensitive nature of questions related to race, identity, and therapeutic practice, participants were explicitly informed that they could skip any item they did not feel comfortable answering.

## Results

### Sample characteristics and clinical context

Most therapists (89.8%) were currently working with children and adolescents, and a further 5.1% had done so within the past year. Most therapists had experience with clients aged 13–17 years (79.6%), followed by children aged 7–12 years (60.2%), and fewer with young adults (34.7%) or children aged 0–6 years (17.3%). A significant portion of therapists worked with clients identifying as BIPoC (64.3%), with a smaller proportion (42.9%) indicating specific experience with clients from Black ethnic groups. However, most therapists estimated that BIPoC clients made up less than 25% of their patient population (90.8%). Regarding gender diversity among clients, 93.9% of therapists had worked with girls/female-identifying clients, 85.7% with boys/male-identifying clients, and 61.2% with non-binary youth. A quarter (24.5%) indicated direct experience working with clients identifying outside the gender binary.

### Prevalence of art-based techniques use

Just over half of participants (51.0%) reported using art-based methods exclusively, while 8.2% used music-based techniques exclusively. An additional 11.2% reported using both modalities, and 29.6% had no prior experience with either. Among those using art-based approaches, 12.2% reported using them “often” and 9.2% “very often,” while the majority applied them “sometimes” (29.6%) or “rarely” (11.2%). Reported use was more frequent in individual (Mean = 3.08, *SD* = .759) than group (Mean = 1.64, *SD* = 1.29) or family settings (Mean = 1.28, *SD* = 1.067). Only 44.9% of therapists provided a diagnosis-specific response, while over half of participants either skipped the question or indicated that creative methods were not tied to particular disorders. Several explicitly reported using creative techniques “independently of diagnosis” or whenever nonverbal expression was needed. Among those who specified indications, emotional dysregulation and affect processing were most frequently mentioned. Anxiety disorders and ADHD were the most commonly named diagnostic categories, followed by depression and trauma-related symptoms. Eating disorders and childhood developmental or communication difficulties (e.g., mutism) were reported less often.

### Correlates of art-based technique use

When including those who used art-based methods either alone or in combination with music-based approaches, 61 of 98 therapists (62.2%) reported using art-based techniques overall. Therapists who worked with BIPoC clients were significantly more likely to use art-based methods (75.4%) compared to those without such experience (45.9%), *χ*²(1) = 8.71, *p* = .003**,** p_FDR = .014, V = .30**.** Similarly, therapists reporting experience with non-binary clients were more likely to use art-based techniques, *χ*²(2) = 18.26, *p* < .001, p_FDR = .009, *V* = .43. In contrast, no association was found between CBT orientation and the use of art-based techniques (*p* = .343). Therapists using art-based methods reported higher levels of self-reflection regarding their social position (*U* = 797, Z = −2.50, *p* = .013, p_FDR = .023, r = .25). Therapists working with children and adolescents were also more likely to report using art-based techniques overall, *χ*²(1) = 4.18, *p* = .041, p_FDR = .046, *V* = .21.

### Training and racism-informed preparation

While many therapists reported working with racially diverse client groups, few had received formal training related to racism-informed care. Only 17.3% reported any training on racism-informed psychotherapy, and an even smaller number (4.1%) had specific training on racial trauma. Among those trained, most described their training as basic (64.7%) or advanced (29.4%). A subgroup analysis of therapists who self-identified as ethnic/racial minorities (*n* = 12) revealed a slightly higher proportion working with BIPoC (66.7%) and Black clients (83.3%). However, access to racism-informed training within this subgroup was also limited.

### Descriptive overview of reported clinical functions

Therapists rated art-based methods as particularly helpful for fostering emotional expression and strengthening therapeutic relationships, especially among children aged 7–12 years. Nearly a third (30.6%) identified this age group as especially suited for emotional work through creative means, while 40.8% found art-based techniques most effective for building therapeutic alliance in the same group. Therapists also noted specific benefits for working with emotions like anger, sadness, and fear, particularly in cases of trauma, anxiety, and ADHD. No gender group effect was reported regarding the perceived usefulness of creative methods, though some participants highlighted a preference for such approaches among female clients (Table 3).

**Table 3:** descriptive data regarding motives, therapeutic goals, and other art-based experiences among different client groups.

Domain	Indicator	General Population (*n* = 61)	PoC Groups (*n* = 35)	Black Ethnic Groups (*n* = 20)
Motives for Use (% endorsed)	Emotional encouragement	91.8	80.0	75.0
	Supplement to therapy	67.2	54.3	50.0
	Non-verbal communication for difficult experiences	57.4	40.0	45.0
	Flexible/individualized	52.5	54.3	50.0
	Self-efficacy	73.8	60.0	70.0
	Therapeutic relationship	73.8	57.1	70.0
	Trauma processing	26.2	14.3	5.0
	Intercultural/cultural identity	3.3	11.4	25.0
Therapeutic Goals (% endorsed)	Emotional regulation	67.2	62.9	65.0
	Trauma processing	26.2	28.6	20.0
	Communication improvement	39.3	45.7	35.0
	Self-reflection/esteem	64.9	60.0	60.0
	Social skills	23.0	20.0	25.0
	Creativity	57.4	45.7	25.0
	Intercultural identity work	3.3	11.4	15.0
	Racial stress/intergenerational trauma^a^	–	2.9–5.7	5.0–10.0
Confidence	Median (IQR)	3.00 (1.00)	3.00 (2.00)	3.00 (1.75)
Engagement	Median (IQR)	4.00 (0)	4.00 (1.00)	4.00 (1.00)
Client Response	Mostly positive (%)	78.7	74.3	65.0
Perceived Effectiveness	Median (IQR)	4.00 (0)	4.00 (1.00)	4.00 (1.00)
Perceived Preparedness	Median (IQR)	2.00 (1.00)	2.00 (1.00)	2.00 (0.00)

a Summarized of more than one question.

### Within-therapist differences across client contexts

To explore whether therapists positioned art-based techniques differently depending on client context, paired within-therapist comparisons were conducted. Within-therapist comparisons between general and PoC client contexts revealed no significant differences in perceived effectiveness, client engagement, or therapist confidence when using art-based techniques (all *p* > .15). However, therapists reported significantly lower preparedness when working with PoC clients (*Z* = −2.63, *p* = .009, p_FDR = .020, *r* = .45). An exploratory Friedman test comparing general, PoC, and Black client contexts (*n* = 18) indicated differences in perceived preparedness, *χ*²(2) = 9.56, *p* = .008, p_FDR = .020, *W* = .27. Preparedness ratings were highest for general clients and lower in minoritized contexts. Given the small subgroup size, these findings should be interpreted cautiously and considered preliminary and hypothesis-generating. Exploratory McNemar tests further suggested shifts in the reported use of art-based activities across client contexts. Therapists appeared less likely to endorse art-based activities for relationship-building purposes in PoC contexts (*p* = .039, p_FDR = .046, OR = 5.0), with more therapists discontinued endorsing relational goals in PoC contexts (*n* = 10) than newly adopted them (*n* = 2). A similiar pattern emerged fort he use of art-based activities to support nonverbal of difficult-to-express experiences (*p* = .021, p_FDR = .032, OR = 9.0), with more therapists discontinuing (*n* = 9) than newly adopting (*n* = 1) this function in PoC contexts. However, given the small cell frequencies and exploratory nature of these analyses, these findings should be interpreted with substantial caution. The PoC item explicitly referenced experiences such as racism.

### Qualitative findings: perceived strengths, challenges, and barriers

Open-ended responses provided insight into therapists' experiences with art-based techniques, including perceived benefits, challenges in implementation, and reasons for non-use. Across responses, a consistent thematic structure emerged comprising (a) barriers to use, (b) perceived therapeutic strengths, and (c) practical challenges in clinical application. The categories derived from the qualitative content analysis are summarized in Table 4.

**Table 4:** Thematic categories derived from open-ended responses on art-based techniques in psychotherapy.

	Main Themes	Definition	Example
Reported Reason not Using Art-based Activities	Lack of formal training or familiarity	The therapist reports insufficient knowledge, education, or training regarding art-based methods and therefore does not feel able to apply them in psychotherapy.	*“Lack of experience with the systematic use of these activities.”*
	Infrastructural constraints in training	Art-based techniques are not used because the psychotherapy training context, supervision, or institutional framework does not include or support their implementation.	*“I am in training and there is no framework for it.”*
	Mismatch with therapy approach	Therapists perceive art-based activities as inconsistent with their primary therapeutic orientation, modality, or professional role.	*“Supervisors rather require classical (including third-wave) behavioral therapy.”*
	Low personal afffinity to/ confidence with art	Non-use is explained by low therapist self-confidence, low perceived artistic ability, or discomfort with using artistic media.	*“I am not a good drawer myself.”*
	Client suitability	Therapists report that art-based activities were not used because they perceived the method as unsuitable for their clients or had insufficient opportunity (e.g., age, preferences, limited exposure).	*“The client was over 18 and did not feel like it.”*
	Resource limitations	Non-use is attributed to practical or organizational barriers such as missing materials, preparation effort, or logistical demands.	*“Lack of materials.”*
Strengths of Art-based Activities	Alternative communication channel	Art-based activities provide a nonverbal or additional mode of communication that enables expression when verbal language is limited, inhibited, or insufficient.	*“Where words are lacking, one can communicate through creative techniques.”*
	Emotional expression and regulation	Art-based methods facilitate the expression, processing, and regulation of emotions that are difficult to access or verbalize.	*“Better possibilities for expression”*
	Increased motivation, participation and transfer	Creative activities increase engagement in therapy and help patients remember and apply therapeutic content in everyday life.	*“Inherent activation of resources and easier recall of the exercise (transfer into everyday life via copy/symbol)”*
	Access to difficult topics	Art-based activities help patients approach distressing, sensitive, or avoided topics more easily.	*“A more playful way of dealing with also distressing topics.”*
	Strengthening self-concept, cultural identity, and agency	Creative work promotes self-efficacy, confidence, identity development, and a sense of personal agency.	*“Individual design possibilities to express oneself, discover/try out one’s own abilities, experience self-efficacy”*
	Improved therapeutic alliance	Art-based activities support rapport building, trust, and collaborative engagement between therapist and patient.	*“Less highly psychologized approach; easier to enter the therapeutic relationship via another topic”*
	Processing and reflection	Art enables deeper self-reflection and supports cognitive and emotional processing of experiences.	*“Free expression; insights into intrapsychic processes; positive influence on transfer into everyday life”*
	Making abstract internal states concrete	Internal experiences such as feelings, memories, or thoughts become tangible and easier to understand through visual representation.	*“Better expression of inner processes”*
	Enjoyment	Patients experience creative activities as pleasant, playful, or fun, which positively influences the therapy atmosphere.	*“A more playful way of dealing with also distressing topics”*
	Developmental suitability	Art-based activities are particularly appropriate for children or developmentally younger clients.	*“Especially younger patients worked more motivated.”*
Challenges of Art-based Activities	Performance anxiety and shame	Patients feel insecure, embarrassed, or ashamed about their artistic abilities and fear negative evaluation of their work.	*“Clients’ fear of not being creative/good enough.”*
	Low motivation/resistance	Patients are reluctant to engage in art-based activities, refuse participation, or show little willingness to try the exercises.	*“Some young people find it difficult to engage”*
	Perfectionism	Patients focus excessively on producing a “good” or aesthetically pleasing product, which interferes with therapeutic processing.	*“Perfectionism; focus on outward appearance of the artwork instead of inner processes.”*
	Infrastructure constraints	Practical limitations such as session length, preparation time, materials, or therapy setting hinder the use of art-based methods.	*“Often time-intensive, therefore double sessions used.”*
	Emotional avoidance	Patients use the activity to avoid emotional processing or disengage from distressing internal experiences.	*“Avoidance tendencies with aversive memories”*
	Developmental and cognitive factors	Age, attention span, cognitive abilities, or symptom-related characteristics affect the feasibility of art-based work.	*“Short attention span — incorporating breaks.”*
	Therapist insecurity	The therapist feels uncertain about how to guide, interpret, or therapeutically use the creative activity.	*“My instructions were sometimes too vague; 50 min sessions were often insufficient”*
	Methodological knowledge	The therapist lacks concrete techniques, instructions, or knowledge of interventions for implementing art-based activities.	*“Finding suitable ideas — literature, intervision.”*
	Maintaining therapeutic focus	The activity risks distracting from therapy goals or dominating the session without sufficient therapeutic processing.	*“Not losing the therapeutic focus.”*
	Translating art into therapy / Integrating into CBT structure	Difficulties linking the creative activity to therapeutic processing, reflection, or treatment goals, including integrating art-based work into structured therapeutic approaches (e.g., CBT).	*“Uncertainty how to use appropriately for other treatment goals”*
	None	The therapist explicitly reported no experienced difficulties when using art-based activities.	–

Categories are presented in descending order of prominence within the qualitative material. Frequencies per category are not reported because individual responses could contain multiple coded themes and varied substantially in length. Example quotations are illustrative and anonymized; they are not linked to individual participants due to the large sample size and to preserve confidentiality.

Across therapists, reported barriers to using art-based techniques primarily concerned training and structural factors rather than negative attitudes toward the methods. Non-use was most commonly attributed to lack of formal training, absence of support within psychotherapy training structures, or perceived incompatibility with therapists’ primary treatment orientation. Personal confidence and client-related factors were mentioned less frequently, and practical resource limitations were rare.

When used, art-based techniques were consistently described as facilitating nonverbal communication, emotional expression, and access to difficult topics. Therapists further reported increased motivation, improved therapeutic engagement, and strengthening of self-concept and self-efficacy. Reported challenges mainly related to patient engagement, particularly performance anxiety and hesitation toward creative tasks, as well as practical constraints such as time and session structure.

### … for PoC clients

Regarding clients of Color, therapists most frequently highlighted the usefulness of art-based techniques for facilitating nonverbal expression and overcoming language-related barriers (*n* = 7). Additional perceived benefits included strengthening self-efficacy and self-worth (*n* = 6), supporting emotional access and insight (*n* = 4), and facilitating relationship building (*n* = 4). Some therapists also described a culturally meaningful or resonant quality of creative work (*n* = 3).

However, several respondents explicitly emphasized that these advantages were not specific to clients of color but comparable to those observed with other client groups (*n* = 8).

Most therapists reported no specific challenges when using art-based activities with clients of color (*n* = 12). Where difficulties were mentioned, they primarily concerned therapist-related limitations (e.g., lack of training; *n* = 3) and general therapeutic process factors such as motivation or avoidance (*n* = 4), rather than culturally specific barriers.

### … for Black clients

For Black clients, reported strengths largely mirrored those described for other groups. Therapists noted facilitated communication and more relaxed interaction (*n* = 4), improved therapeutic relationship (*n* = 2), and benefits for identity development and self-worth (*n* = 2). However, many therapists indicated that they lacked sufficient experience to differentiate effects specifically for Black clients or perceived them as comparable to other client groups (*n* = 7).

Similarly, most therapists reported no specific challenges in the use of art-based techniques with Black clients (*n* = 10). The few reported difficulties were again related to limited training (*n* = 2) or general hesitancy toward creative engagement (*n* = 1).

### Reasons for non-use in PoC and Black clients

Reported reasons for not using art-based techniques were highly similar across both groups. The most common explanation was lack of opportunity to apply such methods, including limited exposure to clients from these groups, short treatment duration (e.g., assessment phases), premature termination, or contextual constraints within clinical settings. A second major reason concerned limited training and perceived competence in art-based methods. Therapists frequently reported not having received formal training, feeling uncertain in their application, or primarily identifying with other therapeutic modalities, particularly music therapy.

Client-related and contextual factors were mentioned less frequently, including perceived mismatch with therapeutic goals, limited client motivation, or use of creative methods only within diagnostic procedures.

## Discussion

This mixed-methods study examined how psychotherapists use art-based techniques in routine practice and whether their application differs when working with minoritized clients. Across quantitative and qualitative findings, therapists consistently described creative methods as helpful in therapy settings, particularly for communication, emotional access, and engagement. However, whether they used art-based techniques in therapy was shaped less by attitudes toward the methods and more by structural factors, including training opportunities, therapeutic orientation, and uncertainty in cross-cultural therapeutic situations.

### Therapist demographics and experience

The sample was largely composed of White, cis-female therapists, consistent with prior descriptions of demographic homogeneity in German psychotherapy workforces (30, 35). Many participants reported working with diverse client populations while simultaneously indicating little preparation for discussing racism or cultural identity. This pattern was further corroborated by qualitative responses, in which barriers to use with PoC and Black clients were predominantly attributed to limited training and perceived competence rather than negative attitudes toward the methods themselves — consistent with the quantitative finding that preparedness, rather than confidence or perceived effectiveness, differed most markedly across client contexts. In exploratory analyses, this was further reflected in a reduced endorsement of art-based techniques for relational purposes and for addressing difficult-to-express topics among PoC and Black clients — findings that should be considered hypothesis-generating.

These findings are consistent with prior work describing therapist hesitancy in engaging race-related topics (36), and with psychotherapy process research suggesting that clinicians frequently experience self-doubt and a preoccupation with “doing the right thing” in complex or emotionally charged encounters (37), particularly early in professional development or in situations perceived as insufficiently prepared for (38). Limited diversity within the workforce may further influence therapists' perceived confidence when addressing identity-related or racialized experiences, particularly in the absence of formal training (24). Work involving unfamiliar identity-related experiences may represent one such context of heightened uncertainty.

One possible interpretation is that therapists who feel uncertain in identity-related contexts may be less inclined to explicitly frame creative techniques as tools for engaging emotionally or socially charged material, and may instead position them as structured therapeutic tasks that provide predictability and containment. This could help explain why relational and emotionally exploratory motives were less frequently endorsed in structured survey items, even though therapists qualitatively described communication and connection as outcomes of the work — though this interpretation remains speculative. Taken together, these patterns tentatively suggest that creative methods may currently function not only as expressive tools, but also as structured entry points in situations where therapists feel uncertain about initiating identity-related dialogue, and that psychotherapy training programs could more systematically address both culturally responsive practice and the clinical use of creative interventions.

### Alliance formation across client contexts

In exploratory analyses, therapists less frequently indicated relationship-building as a reason for using creative methods with clients of Color — a finding that should be treated with caution given the small cell frequencies involved. Notably, quantitative ratings of client engagement remained similarly high across general and PoC client contexts (Table 4). Although relationship-building was less often endorsed as an explicit motive, therapists qualitatively described improved communication, mutual understanding, and connection, including overcoming language barriers.

This pattern is consistent with research suggesting that creative work may facilitate alliance indirectly by providing a shared focus of attention — therapist and client engage together in an activity — which can reduce interpersonal pressure and support connection (39, 40). Such structured, collaborative engagement may be particularly helpful in intercultural therapeutic contexts, and aligns with research on cultural humility highlighting the importance of an other-oriented and respectful stance in strengthening working alliance and promoting therapeutic improvement (41).

This does not necessarily imply a weaker therapeutic alliance. Rather, one possible interpretation is that this pattern points to a distinction between how creative techniques are framed and how they function in practice — suggesting that art-based techniques may support alliance formation not necessarily through explicit relational intention, but through the interactional structure they create. This remains a tentative interpretation that warrants further investigation.

### Transdiagnostic use of creative methods

The minority of therapists report diagnosis-specific applications, most commonly in anxiety disorders, ADHD, depression, and trauma-related conditions. However, therapists' descriptions of indications revealed that art-based techniques were applied across symptom presentations to facilitate emotional access, communication, and experiential processing. At the same time, a discrepancy emerged between quantitative and qualitative findings regarding their use with minoritized clients: while relational motives were reported less frequently for PoC clients in structured items, open-ended responses emphasized their role in nonverbal communication, language mediation, and culturally sensitive engagement.

This transdiagnostic pattern of use aligns with psychotherapy models that focus on shared mechanisms across disorders. The Unified Protocol conceptualizes emotional disorders as maintained by maladaptive responses to internal emotional experiences and therefore targets processes such as emotional awareness, avoidance, and tolerance of affective and bodily states (42, 43). Conceptually, creative arts therapies may provide experiential and symbolic pathways through which similar processes can be addressed. Research on therapeutic factors in creative arts therapies describes embodiment, concretization, and symbolism as mechanisms by which internal experiences can be externalized, explored, and reintegrated (1). Through the transformation of internal states into sensory and symbolic forms, clients may approach emotionally meaningful material indirectly, potentially reducing avoidance while maintaining psychological safety.

This suggests that creative methods may not be exclusively conceptualized as disorder-specific interventions, but are rather flexibly integrated into both diagnosis-oriented and process-oriented therapeutic frameworks — functioning as an alternative therapeutic channel through which psychological material can be explored, particularly when verbal processing is limited. The discrepancy between quantitative and qualitative findings in minoritized client contexts does not suggest reduced relevance of creative methods but rather a difference in how therapists position them within therapy. One possible interpretation is that clinicians may feel less confident introducing creative techniques explicitly as relational interventions in cross-cultural contexts and instead frame them as structured therapeutic activities.

### Convergent findings across methods

Table 5 presents a joint display of quantitative and qualitative findings across four key themes with explicit mixed-methods meta-inferences; a full version with extended descriptions is provided in Supplementary Table S3. Across both data strands, two particularly coherent patterns emerge. First, quantitatively lower preparedness in PoC and Black client contexts, combined with qualitative accounts attributing barriers predominantly to training gaps rather than negative attitudes, converges on the meta-inference that structural underpreparedness may be an important obstacle to identity-informed creative practice. Second, the combination of lower relational motive endorsement in PoC contexts and equally high engagement ratings, alongside qualitative descriptions of improved communication and connection, suggests that art-based techniques may support the therapeutic relationship through the shared activity they create, even when therapists do not explicitly use them with relational goals in mind.

**Table 5:** Joint display: convergent findings across methods.

Theme	Quantitative Finding	Qualitative Finding	Meta-Inference
Prevalence & correlates	Therapists with BIPoC experience more likely to use art-based methods (75.4% vs. 45.9%, *p* = .003)	Nonverbal expression, language mediation, and cultural resonance highlighted as specific benefits	Client diversity experience may drive uptake; mechanisms remain to be examined
Training & preparedness	Significantly lower preparedness in PoC and Black contexts (*p* = .009, *r* = .45)	Barriers attributed to training gaps, not negative attitudes	Structural underpreparedness is the primary obstacle to identity-informed creative practice
Relational framing & engagement	Relational motives less endorsed in PoC contexts (*p* = .039), yet engagement equally high (Median = 4.00)	Improved communication and connection described despite no explicit relational framing	Creative methods may support alliance through shared activity, even without conscious relational intent
Transdiagnostic use	55.1% reported no diagnostic tie; emotional dysregulation most common indication	Used across presentations for emotional access and communication, “independently of diagnosis”	Creative methods function as process-oriented rather than disorder-specific interventions

A full version of this joint display with extended descriptions is provided in Supplementary Table S3.

### Clinical implications

The findings highlight the potential of creative modalities as culturally responsive therapeutic tools, consistent with emerging literature on creative interventions in diverse youth populations (26). Because creative activities allow communication beyond verbal language, they may be particularly suited to psychotherapy with heterogeneous and multilingual youth populations. In routine practice, therapists frequently used creative techniques to facilitate emotional expression, access to avoided topics, and engagement, suggesting that these methods may serve as practical tools for working with developmental, communicative, or emotional barriers.

Importantly, therapists in this study applied creative methods largely independent of diagnosis. This pattern supports a process-oriented perspective in which creative techniques function not primarily as disorder-specific interventions but as experiential approaches targeting emotional awareness, avoidance, and meaning-making. Creative activities may therefore complement structured psychotherapies by offering an alternative channel for emotional processing, particularly when cognitive-verbal interventions are difficult to implement.

The results also have implications for the therapeutic relationship. Therapists frequently described improved engagement, communication, and mutual understanding during creative work, even when relationship-building was not their primary intention. Creative activities may support alliance formation by providing a shared focus of attention and reducing interpersonal pressure, which can be especially helpful with younger clients or in intercultural therapeutic encounters. In this sense, creative techniques may function as relational facilitators in therapy settings.

At the same time, the study revealed a consistent gap between client diversity and therapist preparation. Many clinicians reported working with BIPoC youth but had little training in racism-informed or culturally responsive psychotherapy. Lower perceived preparedness in minoritized contexts may suggest that therapists feel less confident using art-based methods relationally in cross-cultural encounters. Without adequate training, therapists may therefore underutilize their relational and cultural potential.

These findings indicate that training programs should not only introduce art-based techniques but also provide procedural guidance on how to integrate them into case conceptualization, therapeutic dialogue, and identity-informed care. Training in culturally responsive and racism-informed psychotherapy may be particularly important to help clinicians use creative approaches as tools for relational and culturally attuned engagement. Integrating creative methods into psychotherapy training could therefore simultaneously strengthen therapeutic alliance, engagement, and responsiveness to diverse client experiences.

### Theoretical contributions

The present study makes several contributions to existing theoretical frameworks. First, with respect to psychotherapy process theory, the findings suggest that art-based techniques may function not only as expressive tools but potentially also as interactional structures through which therapeutic processes (e.g., emotional access and engagement with charged material) may be facilitated in a less confrontational way. This extends process-oriented accounts of how non-verbal and experiential modalities operate within the therapeutic relationship (39, 40). Second, with respect to multicultural counseling, the study adds practice-based evidence to theoretical accounts of how therapist preparedness and training shape cross-cultural therapeutic encounters (24, 36). The tentative pattern that preparedness — rather than confidence or perceived effectiveness — differed most markedly across client contexts may point to barriers that are more structural than attitudinal in nature, though this interpretation is exploratory and should be tested in future research with larger and more diverse samples. Third, with respect to arts-based intervention science, the transdiagnostic pattern of use observed in this study supports a process-oriented rather than disorder-specific conceptualization of art-based techniques in child and youth psychotherapy — consistent with emerging theoretical frameworks that emphasize shared mechanisms such as embodiment, symbolization, and emotional access as active ingredients of creative interventions (1).

### Strengths and limitations

This study has several limitations. Data were based on therapist self-report and may be influenced by recall or social desirability biases, particularly regarding culturally sensitive topics. In addition, the cross-sectional design does not allow conclusions about causal relationships, for example between training experiences and the use of creative methods. The findings describe therapists' reported practices and perceptions rather than clinical outcomes, and therefore should not be interpreted as evidence regarding the efficacy of art-based interventions. Furthermore, all inferential analyses were bivariate in nature. Future research with larger samples should employ multivariate logistic regression or mixed-effects models to disentangle the independent contributions of therapist and contextual characteristics on art-based technique use, and to account for potential confounding. Another limitation concerns the relatively small sample sizes in several subgroup analyses, particularly analyses involving Black clients or therapists with direct experience using specific creative modalities in these contexts. Some inferential analyses were therefore based on small cell frequencies and limited statistical power, increasing the risk of unstable estimates and both Type I and Type II errors. These findings should consequently be interpreted as exploratory and hypothesis-generating rather than confirmatory. Future studies with larger and more diverse samples are needed to examine subgroup-specific patterns more robustly. Furthermore, the data reflect only therapists' perspectives. The views and experiences of clients, particularly minoritized youth, were not assessed and may differ from clinician reports. Future research should therefore include patient-reported experiences and outcomes. The survey relied on retrospective estimates of routine practice, which may not fully correspond to actual session behavior. In addition, although examples were provided in the survey, the term “art-based techniques” may have been interpreted broadly by participants and thus captured a range of heterogeneous practices and levels of structure. Another limitation concerns the survey instrument itself. As no existing measure adequately captured the study's combined focus on creative techniques, identity-informed practice, and work with racialized client groups, most items were specifically developed for this exploratory study. Although the questionnaire was developed collaboratively within an interdisciplinary expert team and iteratively reviewed for conceptual relevance and clarity, no formal pilot testing or psychometric validation was conducted prior to data collection. The findings should therefore be interpreted with caution, and future research is needed to establish the instrument's reliability and validity. The sample was drawn largely from therapists in training within a specific national training context, which may limit generalizability. An additional limitation concerns the use of racial and ethnic umbrella categories within the German context. The survey relied on terms such as “PoC,” “BIPoC,” and “Black,” reflecting contemporary anti-racist and psychosocial discourse in Germany rather than detailed ethnic or migration-related classifications. While these categories aimed to capture therapists' perceived work with racially minoritized clients, they cannot fully represent the heterogeneity and contextual specificity of racialized experiences within Germany. At the same time, race-related terminology remains inconsistently used and discussed within German psychotherapy research. The present study therefore aimed to use and contextualize these terms carefully and reflexively rather than avoid race-related terminology altogether.

At the same time, the study also has notable strengths. First, it provides insight into routine clinical practice, contributing practice-based evidence on how creative techniques are actually implemented in psychotherapy. Second, including therapists in training allowed examination of how such methods are learned and integrated during the formation of clinical routines, thereby identifying training-related barriers at an early professional stage. Third, the mixed-methods design enabled comparison between structured responses and open-ended accounts, revealing both convergent and divergent patterns that would not have been observable with a single-method approach. In particular, qualitative responses offered context for interpreting quantitative findings and reduced reliance on direct self-evaluations alone. Finally, the study adds data from a German psychotherapy training context, which has been underrepresented in research on creative and culturally responsive psychotherapy practices.

## Conclusions

Overall, the findings indicate that creative, art-based methods are widely valued in child and adolescent psychotherapy and are primarily used to facilitate emotional access, engagement, and communication rather than to target specific diagnoses. However, their application with clients and particularly with minoritized clients appears shaped by clinicians' perceived preparedness and training. Expanding training in both creative methods and culturally responsive psychotherapy may therefore not only reduce barriers to implementation but also allow therapists to use creative modalities more confidently as relational and identity-sensitive therapeutic tools. In doing so, creative approaches may offer an important pathway toward more inclusive and embodied forms of psychological care for diverse youth populations.

## Data Availability

The datasets generated and analyzed during the current study are not publicly available due to the sensitive nature of the data and the potential identifiability of participants, but are available from the corresponding author on reasonable request.
