# Supplementary material for: Patterns of creative technique use in child and youth psychotherapy in Germany: an exploratory mixed-methods survey including identity-informed practice
Source: Front Health Serv. 2026 Jul 7;6:1824536. doi: 10.3389/frhs.2026.1824536 (PMC13385263; doi:10.3389/frhs.2026.1824536)
Supplement: Supplementary file 1 [file Table1.docx]

# **Supplementary Materials**

**Figure S1. Conceptual Framework**


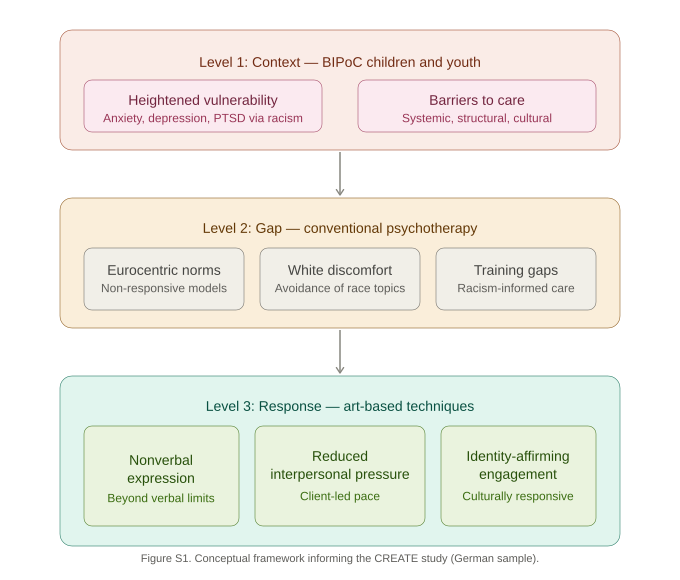


*Figure S1.* Conceptual framework informing the CREATE study (German sample). The framework illustrates three interconnected levels: (1) the heightened vulnerability of BIPoC children and youth and the structural barriers they face in accessing mental health care; (2) the limitations of conventional psychotherapy, including Eurocentric norms, White discomfort, and gaps in racism-informed training; and (3) the proposed role of art-based techniques as a culturally responsive therapeutic response that facilitates nonverbal expression, reduces interpersonal pressure, and supports identity-affirming engagement.

**Supplementary Table S2. Evidence of Measurement Quality**

| **Construct** | **Type** | **N Items** | **α / Convergent validity** |
| --- | --- | --- | --- |
| Motives for Use (general) | Multi-item checklist | 14 | α = .554 |
| Motives for Use (PoC) | Multi-item checklist | 14 | α = .628 |
| Motives for Use (Black) | Multi-item checklist | 14 | α = .565 |
| Therapeutic Goals (general) | Multi-item checklist | 11 | α = .536 |
| Therapeutic Goals (PoC) | Multi-item checklist | 16 | α = .551 |
| Therapeutic Goals (Black) | Multi-item checklist | 16 | α = .490 |
| Confidence | Single-item | 1 | Cross-context rs = .902** |
| Engagement | Single-item | 1 | Cross-context rs = .785** |
| Perceived Effectiveness | Single-item | 1 | Cross-context rs = .670** |
| Perceived Preparedness | Single-item | 1 | Cross-context rs = .839** |

*Note.* For multi-item checklists, Cronbach's alpha is reported as an estimate of internal consistency. For single-item measures, internal consistency is not applicable; cross-context convergent validity coefficients (Spearman's rho) between parallel items across general and PoC client contexts are reported instead. ** p < .001.

**Supplementary Table S3. Joint Display of Quantitative and Qualitative Findings with Mixed-Methods Meta-Inferences**

| **Theme** | **Quantitative Finding** | **Qualitative Finding** | **Mixed-Methods Meta-Inference** |
| --- | --- | --- | --- |
| **1. Prevalence & correlates of art-based technique use** | Therapists with BIPoC client experience were significantly more likely to use art-based methods (75.4% vs. 45.9%, χ² = 8.71, p = .003, V = .30) | Therapists highlighted nonverbal expression, language mediation, self-efficacy, and culturally resonant qualities of creative work as specific benefits for PoC clients; several noted facilitated communication and identity development for Black clients | Direct experience with racially minoritized clients appears associated with greater uptake of art-based techniques, possibly reflecting therapists' recognition of their nonverbal and culturally flexible potential — though the mechanisms underlying this association remain to be examined |
| **2. Training & preparedness** | Only 17.3% reported racism-informed training; therapists reported significantly lower preparedness in PoC contexts (Z = −2.63, p = .009, r = .45), declining further for Black client contexts | Barriers to use with PoC and Black clients were predominantly attributed to limited training and perceived competence rather than negative attitudes; most therapists reported no culturally specific challenges | Structural underpreparedness appears to be the an important obstacle to identity-informed creative practice, with direct implications for training program design |
| **3. Relational framing & engagement** | Relational motives less frequently endorsed in PoC contexts in exploratory analyses (p = .039, OR = 5.0), yet engagement ratings remained equally high across general and PoC contexts (Median = 4.00) | Therapists described improved communication, connection, and nonverbal expression with PoC clients, including overcoming language barriers, despite not explicitly framing creative work as relational | Art-based techniques may support the therapeutic relationship through the shared activity they create — even when therapists do not explicitly use them with relational goals in mind |
| **4. Transdiagnostic use** | The majority of therapists did not report diagnosis-specific applications (55.1% skipped or indicated no diagnostic tie); creative methods most commonly used for emotional dysregulation and affect processing across presentations | Therapists described using creative techniques across symptom presentations to facilitate emotional access, communication, and experiential processing, explicitly noting use "independently of diagnosis" | Creative methods appear to function as process-oriented rather than disorder-specific interventions, supporting a transdiagnostic conceptualization consistent with shared-mechanism models of psychotherapy |

*Note.* Quantitative findings are based on bivariate and within-therapist analyses. Qualitative findings are derived from thematic content analysis of open-ended responses. Mixed-methods meta-inferences represent integrated conclusions drawn from the convergence or divergence of both data strands. Exploratory quantitative findings should be interpreted with caution given small cell frequencies and limited statistical power.
